# A parallel-group randomized controlled trial of a culturally adapted, rumination-focused cognitive-behavioral therapy (RFCBT) guided self-help targeting repetitive negative thoughts in Japanese female university students – study protocol for the RESUME-CBT trial

**DOI:** 10.1186/s40359-026-04182-5

**Published:** 2026-02-16

**Authors:** Yusuke Umegaki, Atsuo Nakagawa, Naoya Todo, Ayako Baba, Dai Mitsuda, Edward R. Watkins, Eugene Mullan

**Affiliations:** 1 Faculty of Human Life and Environment, Nara Women’s University, Kita-Uoya-Nishimachi, Nara, 630-8506 Japan; 2 Department of Neuropsychiatry, St. Marianna University, Kawasaki, Japan; 3 Tokyo Metropolitan University, Hachioji, Japan; 4 Psychology, College of Life and Environmental Sciences, University of Exeter, Exeter, UK

**Keywords:** Rumination, Cognitive behavioral therapy, Repetitive negative thoughts, Depression, Randomized controlled trial

## Abstract

**Background:**

Female university students face an elevated risk of developing common mental health problems, including depression and anxiety. Rumination, a specific form of repetitive negative thought (RNT), is a well-documented risk factor for these conditions. Rumination-focused cognitive-behavioral therapy (RFCBT) represents a promising intervention for the prevention and treatment of depression and anxiety, fostering mental well-being during young adulthood. Although RFCBT has demonstrated effectiveness in numerous randomized controlled trials (RCTs), cultural adaptation has emerged as a critical consideration. This study aims to evaluate the effectiveness of a culturally adapted, guided RFCBT self-help intervention in reducing RNT as well as symptoms of depression and anxiety among Japanese female university students, employing an RCT design.

**Methods:**

This study is a single-site, assessor-blinded, parallel-group, two-arm randomized controlled trial (guided self-help vs. waitlist), employing block randomization in a 1:1 ratio. A total of 102 female university students with elevated levels of RNT will be recruited. Participants will be randomly assigned to either the intervention group receiving guided RFCBT self-help, or the waitlist control group. The primary outcome is rumination, assessed using the Ruminative Responses Scale. Measurements will be conducted at 4- and 8-week post-randomization.

**Discussion:**

Empirical evidence regarding the effectiveness of culturally adapted RFCBT in mitigating RNT and symptoms of depression and anxiety among East Asian populations remains scarce. If proven effective, this study will provide empirical evidence supporting the effectiveness of RFCBT in reducing RNT, depression, and anxiety within an East Asian population.

**Trial registration:**

Japan Registry of Clinical Trials (jRCT): jRCT1050240305, registered 27 March, 2025. (Initially registered with UMIN-CTR: UMIN000053430, on 24 January, 2024. Prospectively registered. Later transferred to jRCT.)

**Protocol version:**

Ver. 1.3, February 9th 2026.

**Supplementary Information:**

The online version contains supplementary material available at 10.1186/s40359-026-04182-5.

## Background

### Young adults as a high-risk population for common mental health problems

Young adulthood, particularly during university years, represents a critical period for mental health development and psychological adjustment, marked by a heightened prevalence of common mental health problems such as depression and anxiety [1–3]. Early onset of these conditions is associated with a greater disease burden [4], as well as poorer academic achievement [5], reduced occupational productivity [6], and difficulties in interpersonal relationships [7]. Japan is not an exception: Japanese university students experience a particularly high prevalence of common mental health problems, often attributed to the difficulties in adapting to university life [8]. Evidence suggests that early interventions are effective in preventing the onset of major depression [9]. The benefits of such interventions are most pronounced when they are directed at young individuals around the onset of mental health problems [3].

### Rumination as a transdiagnostic focus for early intervention

In recent years, interventions targeting transdiagnostic processes that contribute to the onset and maintenance of multiple common mental health disorders, e.g., rumination and avoidance, have been increasingly employed in prevention and treatment (e.g., [10–12]). Rumination is a form of repetitive negative thought (RNT) characterized by a passive and repetitive focus on one’s symptoms, feelings, problems, upsetting events, as well as the circumstances surrounding them and negative aspects of the self [13, 14]. An extensive body of longitudinal prospective studies has demonstrated that rumination predicts the onset, maintenance, and relapse of various mental disorders, including depression and anxiety (e.g., [15–24]). Furthermore, experimental studies provide convergent evidence that rumination causally affects negative mood and thinking (e.g., [25–27]). Accordingly, rumination is recognized as a transdiagnostic risk factor, and is positioned as one of the core elements within the negative valence systems of the National Institute of Mental Health (NIMH) Research Domain Criteria (RDoC) framework.

Moreover, rumination is a symptom that is observed transdiagnostically [28]. In the context of depression, it is frequently present as a residual symptom, with a tendency to remain after remission [29, 30]. Therefore, psychological interventions targeting rumination may hold the potential to address both the underlying mechanisms of depression/anxiety and their symptoms per se.

### Rumination-focused cognitive-behavioral therapy

Multiple randomized controlled trials (RCTs) have demonstrated that cognitive-behavioral therapy (CBT) targeting rumination effectively improves both RNT and symptoms of depression and anxiety [31–33]. Subsequent meta-analyses indicate that the effectiveness of RNT-specific CBT in reducing RNT as well as depressive and anxiety symptoms is comparable to, or even exceeds, that of general CBT approaches [34–36], with reductions in RNT demonstrating a significant association with symptomatic improvement [34, 35]. Given this growing body of evidence, the latest NICE guidelines for adult depression emphasize the significance of targeting rumination to address chronic depressive symptoms and prevent relapse [37].

Rumination-focused cognitive-behavioral therapy (RFCBT; [14]) is a form of CBT designed explicitly to address RNT. The RFCBT employs a functional analytic approach, focusing on the context of rumination – specifically, the antecedent-behavior (rumination)-consequence sequences – rather than focusing on the content of each thought. Clients are encouraged to self-monitor their ruminative response patterns, recognize triggers and early warning signs of RNT, and shift from maladaptive RNT to more adaptive approach behaviors by practicing constructive alternative strategies such as concrete thinking, self-compassion, and absorption. Given that RNT has a causal impact on depression and anxiety, RFCBT is likely to be effective by promoting a direct shift from RNT to more adaptive coping strategies.

The effectiveness of RFCBT in both the treatment and prevention of depression and anxiety has been substantiated through multiple RCTs [31, 33, 38, 39]. For instance, Watkins et al. [33] conducted a phase II trial targeting residual depressive symptoms, comparing the effectiveness of face-to-face RFCBT combined with treatment as usual (RFCBT + TAU) against TAU alone. The results demonstrated substantial between-group effect sizes at post-treatment (*d* = 0.94–1.11), with significantly higher remission rates (RFCBT + TAU: 62%; TAU: 21%) and lower relapse rates (RFCBT + TAU: 9.5%; TAU: 53%). Furthermore, Hvenegaard et al. [31] evaluated the effectiveness of group-based RFCBT versus group-based CBT in patients with depression. After 11 three-hour sessions, their findings indicated the superiority of group-RFCBT over group-CBT (between-group effect size: *d* = 0.38, 95% *C.I.*: 0.03–0.73). Regarding prevention, Topper et al. [39] compared group-based and Internet-based RFCBT with a waitlist control. Significant between-group differences in RNT (intervention conditions versus waitlist control: *d* = 0.53–0.89) and depressive and anxiety symptoms (*d* = 0.36–0.72) were observed at post-intervention, with these effects persisting at the 12-month follow-up. Additionally, Cook et al. [38] reported that guided Internet-based RFCBT self-help significantly reduced the risk of developing depression compared to TAU (*HR* = 0.43), with this protective effect maintained over 15 months. Building upon these findings, a recent systematic review provided evidence supporting the effectiveness of RFCBT in reducing both rumination and depressive symptoms [40].

### Development of a guided RFCBT self-help Japanese version

Nonetheless, the vast majority of such trials have been conducted in Western countries. To the authors’ knowledge, only a single trial has been conducted in an Eastern context to date. In a three-arm RCT comparing the effectiveness of RFCBT and mindfulness-based interventions among a Chinese sample, Mak et al. [41] found no significant differences between the intervention groups and a psychoeducation-only control group in post-intervention reductions in RNT and depression and anxiety symptoms. Although the findings of Mak et al. [41] should be interpreted with caution, particularly in light of relatively high attrition rates, their results highlight the challenges of culturally adapting empirically supported interventions across diverse cultural groups while maintaining fidelity to the original intervention principles [42, 43]. Meta-analytic evidence supports the efficacy of culturally adapted psychological interventions, particularly when delivered to members of the majority ethnic group [44].

To facilitate the cultural adaptation of RFCBT, the authors conducted preliminary case-series studies [45, 46] to develop and assess the feasibility of an RFCBT program tailored for a Japanese population. Umegaki et al. [46] developed an RFCBT guided self-help intervention for female university students with elevated levels of RNT, and examined its preliminary effectiveness using a single-case experimental design. Quantitative findings indicated that the RFCBT-guided self-help program has the potential to reduce both RNT and depressive and anxiety symptomatology at post-intervention, with a subsequent long-term follow-up study indicating that these changes are sustained over a 12-month period [45]. Moreover, qualitative results revealed that participants regarded the program as acceptable and beneficial, although some reported challenges (e.g., difficulty engaging in repeated practice in daily life).

Following refinements and elaboration based on these studies, a culturally adapted RFCBT self-help intervention was developed. This intervention is grounded within the principles of RFCBT, and incorporates specific RFCBT techniques, including self-monitoring, identifying warning signs, If–Then plans, concreteness, absorption, and self-compassion. Additionally, recognizing that self-help programs can have added benefits through cultural adaptation [47, 48], adaptations were implemented based on the following dimensions [49]: (1) language translation, (2) metaphors and content (e.g., following Umegaki et al. [46], we cautiously avoided phrasing that may elicit unnecessary self-criticism during self-monitoring of rumination), (3) concepts (e.g., given that some participants in Umegaki et al. [46] reported difficulty distinguishing self-compassion from self-indulgence, we provided explicit clarification of the distinction between these constructs), (4) goals (e.g., maintaining core RFCBT goals while emphasizing relationship harmony, a foundational basis for cultivating a meaningful sense of self within the Japanese cultural context ([50]), and (5) context (e.g., targeting high-risk female students).

One significant advantage of developing guided self-help interventions is that it facilitates the effective translation and introduction of standardized, empirically-supported psychological treatments. In Japan, the practice of CBT remains limited, with a relatively low number of practitioners delivering CBT [51]. Guided self-help provides materials grounded in empirically-supported interventions, ensuring high fidelity to the original treatment principles and methodologies.

So far, the effectiveness of RFCBT has been evaluated in various formats, including face-to-face sessions [33], group settings [31], and guided online self-help [38, 39, 41]. However, guided self-help delivered in a workbook format has not yet been examined. Compared to online self-help, workbook-based guided self-help may reduce distractions associated with Internet use, reach populations with infrequent or limited Internet access or digital literacy, and address privacy and security concerns more effectively.

### The current study

The present study aims to evaluate the effectiveness of a culturally-adapted, workbook-based RFCBT guided self-help intervention within an RCT design. Our study, the RESUME-CBT (Resilience through Emotional Support for University Mental well-being and Empowerment through CBT) trial, is a single-site, assessor-blinded, parallel, two-arm, block randomized controlled trial, assessing the effectiveness of culturally-adapted RFCBT guided self-help in reducing RNT and symptoms of depression and anxiety post-intervention, compared to a waitlist control. A waitlist control group, which attempts to control for the passage of time and repeated assessment in the target population [52], was implemented to address the methodological limitations of our earlier studies. In those investigations, the effectiveness of the Japanese version of RFCBT guided self-help had been evaluated solely within a single-group design [45, 46], thereby limiting our ability to disentangle true intervention effects from spontaneous improvements or regression to the mean (but see Furukawa et al. [53], for evidence indicating that waitlist groups may represent a suboptimal control condition).

Given that RNT is an established risk factor, this trial examines whether guided RFCBT self-help intervention can reduce RNT among young Japanese adults at high risk for RNT (i.e., female university students), and thus reduce symptoms of depression and anxiety. In other words, our trial serves as a proof-of-concept study exploring the potential of RFCBT in targeting rumination and worry within a Japanese population.

Additionally, we will investigate whether changes in RNT predict subsequent changes in symptoms of depression and anxiety. A recent meta-analysis suggests that rumination-focused CBTs reduce rumination, thereby alleviating depressive symptoms [35]. However, data are insufficient to conclude definitively that rumination-focused CBTs reduce depression (and/or anxiety) by decreasing RNT. This study thus aims to address this evidence gap.

This study will focus on Japanese female university students (aged 18–30 years) with a high tendency toward RNT for several compelling reasons. First, Japanese female university students are identified as having the highest risk of experiencing depression [8]. Furthermore, evidence suggests that rumination is a particularly strong predictor of depression among females (e.g., [54]), emphasizing the elevated vulnerability to depression and anxiety in young female adults. In conclusion, addressing the mental health risks and promoting the well-being of young Japanese female adults, who face heightened susceptibility to depression and anxiety, is of critical importance.

### Hypotheses

Our hypotheses are as follows:

- *Primary Hypothesis*: RFCBT guided self-help will demonstrate superiority over the waitlist control in reducing self-reported rumination (measured by the Ruminative Responses Scale [55]), as assessed at 4-week and 8-week post-randomization.
- *Secondary Hypothesis*: RFCBT guided self-help will demonstrate superiority over the waitlist control in reducing self-reported worry, depression, and anxiety (measured by the Penn State Worry Questionnaire [56], Patient Health Questionnaire-9 [57], and the Generalized Anxiety Disorder-7 [58]), as measured at 4-week and 8-week post-randomization.

## Methods

This trial will be conducted and reported in accordance with the CONSORT 2025 statement [59]. See Appendix 1 for the SPIRIT 2013 Checklist [60].

### Trial design

This is a single-university, assessor-blinded, parallel, 2-arm, block randomized (1:1) controlled superiority trial for female university students exhibiting high levels of RNT, conducted in Nara, Japan. To maintain a 1:1 allocation ratio between the two arms, block randomization will be applied, with each set of two participants randomized to one of the two conditions.

### Participants and eligibility criteria

Participants in this study are female university students (recruited from a single national university in Japan) aged 18–30 years, with fluent proficiency in Japanese communication. Participants are recruited through university courses and classes, as well as through the distribution of flyers and posters posted on campus. Upon completion of each workbook, participants will receive compensation in the form of a library card valued at 1,000–1,500 JPY (approximately 6.46–9.69 USD).

#### Inclusion criteria

This trial employs a selective approach [61], recruiting high ruminators or worriers. Participants are identified as high ruminators or worriers if they score at or above one standard deviation above the mean on the Ruminative Responses Scale (RRS) (≥ 55; [62]), or on the Penn State Worry Questionnaire (PSWQ) (≥ 64, [63]).

#### Exclusion criteria

Given that the focus of this study is not on treatment, students will be excluded if they (a) exhibit high levels of depression, defined as a score ≥ 22 on the Patient Health Questionnaire-9 (PHQ-9) (corresponding to ≥ 90% specificity for a current major depressive episode; [64]), or (b) report ≥ 2 on item 9 (suicidal ideation) of the PHQ-9. Additionally, students receiving regular psychiatric treatment or counseling at the time of recruitment are excluded.

### Screening and consent procedure

After receiving a description of the study, students who express interest in participating are requested to contact the trial manager via email. Upon receipt, the trial manager provides an online screening questionnaire, the responses to which are evaluated to determine eligibility. For eligible participants, the trial manager arranges an individual meeting to deliver a comprehensive explanation of the study and obtain written informed consent. Subsequently, the trial therapist informs the participant about their group allocation. For participants allocated to the intervention group, RFCBT guided self-help commences immediately thereafter. During this meeting, participants are reminded of their right to withdraw consent at any time.

### Randomization and concealment

Participants will be randomly allocated to either the RFCBT guided self-help intervention group or waitlist control group in a 1:1 ratio. Randomization will be concealed from the investigators by use of an off-site, computer-based randomization. To maintain a 1:1 allocation balance, block randomization will be employed, such that every two participants are randomly allocated to either the intervention or waitlist condition. All assessments will be conducted by researchers who are blinded to the randomization process. Assessor blinding will be ensured by administering all assessments online and by avoiding any in-person contact with participants after randomization. Follow-up reminders will be systematically scheduled and disseminated in an identical manner across both groups.

### Intervention

**Fig. 1:**
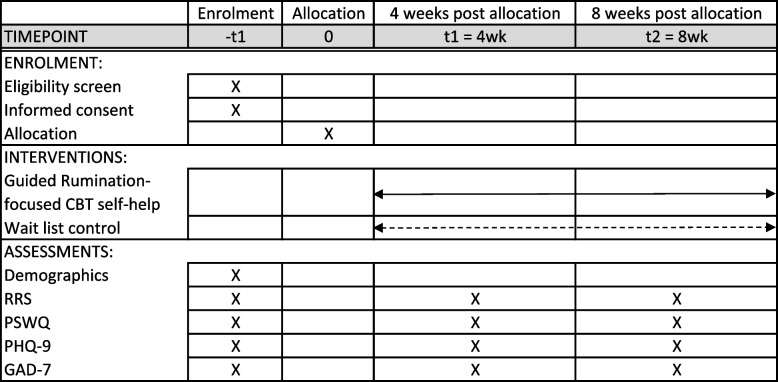
Participant timeline

The participant timeline, in accordance with the SPIRIT 2013 Statement [60], is presented in Fig. 1. The detailed description of the guided RFCBT self-help intervention in TIDieR format [65] is provided in Appendix 3.

#### RFCBT guided self-help

RFCBT is a form of CBT that specifically targets rumination as an overlearned, habitual form of avoidance [14]. RFCBT employs a functional and contextual approach with a specific focus on the sequence of the target behavior, rumination. Through identifying habitual patterns, participants are encouraged to shift to more adaptive processing styles using experiential exercises, imagery exercises, and behavioral experiments. RFCBT introduces specific processing modes as alternative strategies, including concrete thinking, absorption, and self-compassion, which participants are encouraged to explore and adopt.

In this study, we utilized and further refined the Japanese version of RFCBT self-help intervention originally developed by Umegaki et al. [46]. The original intervention consisted of three distinct modules, each presented in a separate workbook, which was likewise implemented in this study. The first module focuses on psychoeducation about rumination and promotes self-monitoring to identify habitual ruminative patterns. Upon identifying these patterns, participants are encouraged to develop plans to respond to cues of stress in an adaptive manner (“If–Then plans”). The second module introduces concrete thinking as an adaptive processing style, and encourages participants to try through behavioral experiments. Concrete thinking is a contextual, specific, and process-oriented mode that contrasts with the abstract, overgeneralized, and decontextualized styles typical of rumination. Standalone concreteness training itself has shown efficacy as an intervention for depression [66]. The third module focuses on cultivating a compassionate processing mode as an alternative to ruminative self-criticism, encouraging participants to practice self-compassionate self-talk and behavior.

As the original intervention omitted one of the core components of RFCBT (the absorption module; [45]), we integrated it as a fourth module. Absorption, a core technique in RFCBT [14], closely resembles the psychological state of “flow” [67], characterized by diminished self-consciousness, concentrated engagement with the task at hand and the immediate present, and a seamless integration of perception and action [14]. This psychological state counteracts rumination, which is marked by heightened self-consciousness, self-criticism, negative evaluation, and conceptual, evaluative thinking. The fourth module promotes the adoption of an absorbed, process-focused state as an alternative to self-analytical rumination. Participants receive psychoeducation on absorption, and are encouraged to recall absorbing memories to foster an engaged state and systematically plan activities that cultivate absorption in daily life.

Each of the four modules is presented in a separate workbook. Each workbook, containing 12–24 pages of text, illustrations, exercises, and checklists, requires approximately one hour to complete, though participants are encouraged to engage with each workbook over 1–2 weeks to thoroughly practice the strategies in their daily lives.

Therapist support will be provided through scheduled, in-person individual sessions with each participant upon completion of each workbook. The trial therapist (YU), a certified public psychologist and clinical psychologist in Japan, has extensive experience in RFCBT training, having completed 1- and 2-day workshops and received individual and group supervision from the RFCBT founder (EW) for both face-to-face cases and online self-help guides, in addition to research and clinical practice. Each individual session, lasting approximately 15–40 min, will focus on reviewing participant progress, encouraging repeated practice of beneficial techniques, addressing any difficulties encountered, and fostering a sense of hope. For each completed workbook, participants will receive a book voucher worth 1,000-1,500 yen as compensation.

#### Waitlist control

The waitlist control group will receive the RFCBT guided self-help program following an 8-week waiting period.

### Outcome measures

#### Primary outcome measure: Rumination

The RRS [55] is a 22-item self-report measure designed to assess the frequency of depressive rumination on a 4-point scale (1: Almost Never to 4: Almost Always). The Japanese version has demonstrated sufficient internal consistency (Cronbach’s α = 0.93), test–retest reliability (*r* = 0.75 over a 6-week period), as well as factorial and concurrent validity (*r* = 0.56–61 with PSWQ, depression [68]), and self-preoccupation [69] scales) [62], and has shown measurement invariance with the original version [70]. The total score across the 22 items will be used as the primary outcome measure. A higher score reflects a greater propensity for rumination.

Additionally, we will use the two subscale scores, i.e., brooding and reflective pondering, as secondary outcome measures, each comprising 5 items from the RRS [55]. Hasegawa [62] reported Cronbach’s α = 0.81 for brooding and α = 0.75 for reflective pondering.

#### Secondary outcome measures: Worry, depression, and anxiety

In addition to brooding and reflective pondering, the following self-report measures will be used as secondary outcome measures.

The PSWQ [56] is a 16-item self-report measure that assesses the respondent’s tendency for worry on a 5-point scale (1: Not at all typical of me to 5: Very typical of me). The Japanese version has demonstrated sufficient internal consistency (Cronbach’s α = 0.92), test–retest reliability (*r* = 0.88 over a 2-week period), and concurrent validity (*r* = 0.72 with State-Trait Anxiety Inventory [STAI]-Trait [71]]) [63]. The total score across the 16 items will be used. A higher score reflects an increased propensity for worry.

The PHQ-9 [57] measures the frequency of depressive symptoms over the past two weeks on a 4-point scale (0: Not at all to 3: Nearly every day). The Japanese version has shown high sensitivity and specificity (between 0.84 and 1.00), as well as high convergent validity (Kappa coefficientκ = 0.79 for diagnosis of major depressive disorder) [72]. The total score of the 9 items will be used. A higher score indicates greater severity of depressive symptomatology.

The Generalized Anxiety Disorder-7 (GAD-7; [58]) assesses the frequency of anxiety symptoms over the past two weeks on the same 4-point scale as the PHQ-9. The Japanese version has demonstrated sufficient sensitivity and high specificity (with a cutoff score of ≥ 10 yielding a sensitivity of 70.6% and specificity of 93.5%,[73]), as well as high concurrent validity (*r* = 0.74 with STAI [71]]; [74]), reflecting robust construct validity. The total score of the 7 items will be used. A higher score reflects increased severity of anxiety.

#### Process measures: RNT and alternative responses as habits

Two self-report measures are incorporated to assess the habitual tendency for rumination and the use of alternative processing modes. To evaluate the habitual tendency for rumination, we will include 12 items from the Habit Index of Negative Thinking (HINT; [75]), translated by the authors for this study. Additionally, the 9 items from the HINT were selected to constitute a separate measure assessing participants’ habitual use of alternative coping strategies.

### Adverse events

The intervention program used in this trial has previously demonstrated feasibility and effectiveness in preliminary studies [45, 46]. The primary aim of the self-help intervention is to enhance psychological resilience by promoting a shift from maladaptive to adaptive processing styles. However, given that the intervention requires participants to engage with and manage stressful situations, the potential for increased risk in certain participants cannot be completely ruled out. Should any adverse event occur, we will adhere to the university’s basic policy for student support, which prioritizes the physical and psychological health and safety of students. In instances where a participant exhibits a substantial increase in depressive symptomatology (e.g., a score exceeding the exclusion threshold) or heightened suicidal ideation (e.g., a score of ≥ 2 on PHQ-9 item 9), they will be promptly informed and strongly encouraged to seek immediate support from the university’s student health service center (https://www.nara-wu.ac.jp/hoken/) or student counseling center (https://www.nara-wu.ac.jp/soudan/). The contact information for these centers is provided at enrollment and reiterated at any time the participant reports an elevated level of risk. If the participant is currently under regular psychiatric care, we will also advise them to consult their treating psychiatrist as soon as possible. The principal investigator will maintain close contact with the participant to ensure that their symptom severity or suicidal risk decreases from a critical clinical level. Additionally, the principal investigator will evaluate whether the participant should continue in the study and assess the need for alternative treatment options, such as initiating or resuming regular psychiatric care. The principal investigator will convey this determination directly to the participant.

### Discontinuations

#### Discontinuation of intervention

If a participant meets any of the following criteria, the trial therapist will decide to discontinue the intervention. The participant will be invited to respond to regular assessments throughout the remainder of the study period.

1. If the participant withdraws consent to continue with the self-help intervention.
2. It the trial therapist considers appropriate to discontinue the study intervention from certain reasons (e.g., emergence of adverse events, serious and imminent ideation of suicide or self-harm, or other severe conditions requiring psychiatric or medical care).
3. If the therapist considers appropriate for the participant to receive intensive face-to-face psychological or psychiatric care.

#### Discontinuation of regular assessment

If the participant withdraws consent to receive study assessment or to take part in the trial, the participant will not be contacted for further assessments and will be considered to have dropped out from the study.

### Sample size estimation

We estimated required sample size using G*power 3 [76]. Umegaki et al. [46] reported RRS mean change of *M* = 18.70 from pre- to post-intervention for RFCBT guided self-help. Therefore, we expect a slightly modest mean change of *M* = 16 for the intervention group, and, *M* = 5 for the waitlist control group (estimated from unpublished longitudinal data, reflecting natural recovery and regression to the mean). Standard deviations (*SD*) of change in RRS varies between intervention and non-intervention samples (*SD* = 14.1, 15.1; [46],unpublished data). We have based our sample size estimate on the pooled *SD* (14.5). Therefore, we estimate a between-group effect size of Cohen’s *d* = 0.75. To detect the effect size of *d* = 0.75 between RFCBT self-help and waitlist control at a two-tailed significance level of 5%, and to obtain 90% statistical power, each condition requires 39 participants. Assuming the dropout rate of 23% [46], we will recruit 51 participants into each condition.

### Statistical analysis

The main analysis will be an intention-to-treat (ITT) analysis, in which analysis is based on allocation, and based on complete case outcome data. For the main analysis, a covariate-adjusted analysis based on a general linear model, or equivalent structural equation modeling, will be conducted. This analysis will examine the primary outcome of between-group differences in rumination (RRS) 4- and 8-week post-randomization, along with secondary outcomes of between-group differences in brooding, reflective pondering, worry, depression, and anxiety. In this analysis, outcome scores at 4- and 8-week post-randomization serve as dependent variables, with measurement timing (4- vs 8-week) and condition (intervention vs control) as independent variables, and baseline scores included as covariates. Additionally, multivariate latent growth models will be used to assess whether changes in RNT predict changes in depression and anxiety. Missing data will be addressed using multiple imputation (MI) or full information maximum likelihood (FIML) method as appropriate. All statistical tests will be conducted at a 5% two-tailed significance level.

### Data collection and management

Research data will be collected in the following forms: (1) Electronic data, which participants will directly input, will be encoded and stored securely in an online database and subsequently transferred to the study database by the research team; (2) paper-and-pencil forms, which will be collected and manually entered into the study database by the trial therapist.

#### Study monitoring

Throughout the course of the study, the Data and Safety Monitoring (DSM) officer (AB) is responsible to conduct periodic inspections of the accumulating outcome and process data. The DSM officer may request additional assessment or follow-up of participants who present increased risk or clinically significant events.

#### Interim analysis

Interim analysis will be performed on the primary and secondary outcome measures when 50% of participants have been randomized and have completed the 8-week follow-up. The interim analysis will be conducted by the DSM officer. Results of the interim analysis will be reported to the authors. The authors will discuss the results of the interim analysis, and decide on the continuation of the trial. The trial may be stopped or modified following inflated risk, as indicated by significant increases in scores of outcome measures or specific risk items (e.g., depression, risk of self-harm/suicide).

#### Auditing

Auditing will be conducted by an independent psychiatrist (AN) and a clinical psychologist (DM) through monthly online meetings with the principal investigator. This process will follow a risk-based approach, emphasizing the monitoring of withdrawal and dropout rates, as well as the occurrence of adverse events.

#### Access to data

The principal investigator, trial manager, and DSM officer will be granted access to the dataset. Additionally, the statistician (NT) will have access to the final dataset to conduct primary analyses.

#### Confidentiality

All study-related data will be securely stored either at the study site or within a protected database. Participants’ information will not be disclosed outside the study without their written permission.

### Ethical considerations and trial pre-registration

The research protocol received approval from the institutional review board of the first author’s affiliated university (approval number 23–32, approved October 26th, 2023; latest version: approval number 25–79, approved December 25th, 2025). This trial has been registered with the jRCT (jRCT1050240305) (initially pre-registered with the UMIN-CTR [UMIN000053430]). Recruitment commenced in January 2024. If significant modifications to the protocol arise, they will be conveyed through amendments to the registered trial information.

### Dissemination policy

The findings of this trial will be disseminated through publication in relevant peer-reviewed journals. Every effort will be made to minimize the interval between the completion of data collection and the dissemination of results.

All individuals who have made significant contributions to the design, conduct, analysis, interpretation, and reporting of this trial will be granted authorship in the final report. Furthermore, authorship of ancillary studies, if any, will be deliberated and determined on a case-by-case basis, following a thorough assessment of each member’s contribution to the study’s design, conduct, analysis, interpretation, and reporting of findings.

To enhance the accessibility of these self-help resources, two of the authors (YU, AN) have published a self-help book in Japanese [77], based on the materials examined in our preliminary studies [45, 46]. Furthermore, the authors will make every effort to ensure that the self-help materials employed in this clinical trial are made publicly available at the earliest feasible stage, either online or in workbook format, while taking into consideration the trial’s implementation status and the planning progress of subsequent clinical trials.

## Discussion

RFCBT employs a functional analytic framework to directly intervene on rumination with the aim of shifting individuals toward more adaptive modes of processing [14]. RFCBT has demonstrated its efficacy across multiple randomized controlled trials [31, 33, 38, 39, 41],however, most trials have been conducted within Western populations. Efforts to adapt RFCBT for distinct cultural contexts while maintaining high fidelity to its foundational principles remain limited.

Accordingly, this single-site, assessor-blinded, parallel, two-arm, block randomized controlled trial seeks to evaluate the effectiveness of a culturally adapted version of RFCBT delivered in a guided self-help format using printed workbooks. We selected Japanese female university students as our target population, given their elevated risk for mental health difficulties [8, 54], and our intention to examine the cross-cultural generalizability of RFCBT. The intervention comprises four workbooks, each incorporating core components of RFCBT: self-monitoring and functional analysis, concreteness, compassion, and absorption. Intervention effects will be evaluated relative to a waitlist control group, enabling us to distinguish intervention-specific effects from spontaneous improvement or regression to the mean.

This study offers a unique opportunity to examine the efficacy of RFCBT in a culturally adapted format within a population whose cultural background differs from those previously studied and for whom intervention effects remain unknown, thereby yielding important clinical implications. Furthermore, evidence supporting the effectiveness of the culturally-adapted RFCBT will substantiate and validate the rigor of our cultural adaptation process.

If rumination is substantially reduced among participants following the intervention relative to the waitlist control, this will indicate that the culturally-adapted RFCBT is an effective intervention for addressing rumination in Japanese female university students. Given the established associations between rumination and both depression and anxiety, demonstrating reductions in rumination would further support RFCBT as a promising intervention and prevention approach for these conditions among Japanese female young adults.

Reductions in RNT following RNT-focused cognitive-behavioral interventions are associated with symptomatic improvement [34, 35]. However, empirical evidence demonstrating that symptom reduction is a consequence of diminished rumination remains limited [35]. The present study will advance theoretical understanding of rumination as a central risk factor for depression and anxiety by examining its role as a psychopathological mechanism that exerts a causal influence on these conditions.

Additionally, it is crucial to explore whether RFCBT can be effectively delivered as a guided self-help intervention through a workbook format. Although RFCBT has been administered via guided online self-help formats [38, 78], no study has yet examined its effectiveness in a workbook format. This study aims to evaluate the effectiveness of this previously untested mode of delivery.

### Strengths and limitations

This study has several limitations. Although RFCBT guided self-help has demonstrated promising results in reducing RNT as well as depressive and anxiety symptoms in Japanese samples, these findings were derived from single-case designs [45, 46]. To delineate true intervention effects from spontaneous improvement or regression to the mean, the present study therefore incorporates a waitlist control condition. While comparisons between the self-help and waitlist conditions enable us to ascertain whether observed effects reflect genuine therapeutic benefit rather than spontaneous remission, and although waitlist controls remain widely used in clinical trials evaluating psychological interventions [52], some evidence suggests that waitlist conditions may exert detrimental effects [53]. Accordingly, once intervention effects are established within our design, future studies may profit from employing no-treatment or non-specific control conditions.

Because our primary objective is to detect intervention-related change, this trial employs a pre-post design that assesses relatively short-term outcomes (4- and 8-week post-randomization). However, to determine whether reductions in RNT meaningfully protect participants from future depressive episodes, longer-term follow-up assessments are essential. Subsequent studies should evaluate whether treatment gains are sustained over extended periods following the termination of the intervention.

Third, our sample size was calculated based on expected between-group differences; thus, additional multivariate latent growth analyses are insufficiently powered. Fourth, the single-site design may increase the risk of sampling bias.

Despite these limitations, the study possesses notable strengths. To our knowledge, this is the first adequately powered RCT to evaluate the effectiveness of RFCBT in reducing RNT in a non-Western population. Prior to the trial, the content and delivery of the guided self-help program were refined and substantiated through preliminary case-series studies [45, 46], enhancing the likelihood that the intervention would be effective in our target population.

University students in Japan, as well as in other countries, are experiencing a mental health crisis. Demonstrating that high-risk students with elevated RNT can be effectively supported would represent a critical step toward improving the mental health of this vulnerable population.

## Supplementary Information

Supplementary Material 1. Appendix 1. SPIRIT 2013 Checklist: Recommended items to address in a clinical trial protocol and related documents.

Supplementary Material 2. Appendix 2. Trial registration data.

Supplementary Material 3. Appendix 3. Description of the intervention.

Supplementary Material 4. Appendix 4. Documentation given to participants and consent form.

## Data Availability

The datasets used during the current study will be available from the corresponding author on reasonable request.

## Abbreviations

RNT: Repetitive negative thoughts
CBT: Cognitive behavioral therapy
RFCBT: Rumination-focused cognitive behavioral therapy
RCT: Randomized controlled trial
DSM: Data and safety monitoring
NIMH: National Institute of Mental Health
RDoC: Research Domain Criteria
TAU: Treatment as usual
NICE: National Institute of Health and Clinical Excellence
RRS: Ruminative Responses Scale
PSWQ: Penn State Worry Questionnaire
PHQ-9: Patient Health Questionnaire-9
GAD-7: Generalized Anxiety Disorder-7
HINT: Habit Index of Negative Thinking
ITT: Intention-to-treat
MI: Multiple imputation
FIML: Full information maximum likelihood
jRCT: Japan Registry of Clinical Trials
NWU: Nara Women’s University
JSPS: Japan Society for the Promotion of Science
