# Supplementary material for: A parallel-group randomized controlled trial of a culturally adapted, rumination-focused cognitive-behavioral therapy (RFCBT) guided self-help targeting repetitive negative thoughts in Japanese female university students – study protocol for the RESUME-CBT trial
Source: BMC Psychol. 2026 Feb 16;14:275. doi: 10.1186/s40359-026-04182-5 (PMC12947469; doi:10.1186/s40359-026-04182-5)
Supplement: Supplementary file 2 — Supplementary Material 2. Appendix 2. Trial registration data. [file 40359_2026_4182_MOESM2_ESM.doc]

*Appendix 2. Trial registration data*

| Item No. | Data Category | Information |
| --- | --- | --- |
| 1 | Primary Registry and Trial Identifying Number | Japan Registry of Clinical Trials (jRCT): jRCT1050240305 |
| 2 | Date of Registration in Primary Registry | March 27th, 2025 |
| 3 | Secondary Identifying Numbers | UMIN-CTR UMIN000053430 (Prospectively registered on January 24th, 2024. Later transferred to jRCT) |
| 4 | Sources of Monetary or Material Support | Japan Society for the Promotion of Science (JSPS); Nara Women's University |
| 5 | Primary Sponsor | Yusuke Umegaki, PhD. Faculty of Human Life and Environment, Nara Women's University |
| 6 | Secondary Sponsor(s) | N/A |
| 7 | Contact for Public Queries | Yusuke Umegaki, PhD. (email: y.umegaki@cc.nara-wu.ac.jp). Faculty of Human Life and Environment, Nara Women's University. Kita-Uoya-Nishimachi, Nara City, Nara, 630-8506 JAPAN. |
| 8 | Contact for Scientific Queries | Yusuke Umegaki, PhD. (email: y.umegaki@cc.nara-wu.ac.jp). Faculty of Human Life and Environment, Nara Women's University. Kita-Uoya-Nishimachi, Nara City, Nara, 630-8506 JAPAN. |
| 9 | Public Title | Resilience through Emotional Support for University Mental well-being and Empowerment through CBT (RESUME-CBT) |
| 10 | Scientific Title | Developing resilience through rumination-focused cognitive-behavior therapy self-help in female university students: Randomized controlled trial |
| 11 | Countries of Recruitment | Japan |
| 12 | Health Condition(s) or Problem(s) Studied | Repetitive negative thoughts (rumination, worry), depression, anxiety |
| 13 | Intervention(s) | Intervention group: Rumination-focused cognitive-behavioral therapy (RFCBT) guided self-help  Control group: 8 weeks Waitlist |
| 14 | Key Inclusion and Exclusion Criteria | Ages eligible for study: 30≥ and ≥18 years; Sexes eligible for study: female  Inclusion criteria: High ruminators/worriers, as measured by the RRS (≥ 55; Hasegawa, 2013) and PSWQ (≥ 64; Sugiura & Tanno, 2000)  Exclusion criteria: (a) Students scoring ≥ 22 on PHQ-9 (Inoue et al., 2012), or ≥ 2 on PHQ-9 item 9 (suicidal ideation); (b) Students who are receiving regular psychiatric treatment or counselling |
| 15 | Study Type | Interventional  Allocation: Block randomized; Intervention model: Parallel assignment; Masking: Single (assessor)-blind  Primary purpose: Prevention  Phase II |
| 16 | Date of First Enrollment | January 2024 |
| 17 | Sample Size | Plan to enroll: 102; Enrolled: 39 |
| 18 | Recruitment Status | Recruiting |
| 19 | Primary Outcome(s) | Outcome Name: Rumination  Method of measurement: Ruminative Responses Scale (RRS) Score  Timepoint: 4- and 8-weeks following allocation |
| 20 | Key Secondary Outcomes | Outcome Name: Worry, Depression, Anxiety  Method of measurement: Penn State Worry Questionnaire (PSWQ), Patient Health Questionnaire -9 (PHQ-9), Generalized Anxiety Disorder -7 (GAD-7)  Timepoint: 4- and 8-weeks following allocation |
| 21 | Ethics Review | Status: Approved  Date of approval: October 26th, 2023  Name and contact details of Ethics committee: Ethics Review Committee on Research with Human Subjects, Nara Women's University [kenkyou@cc.nara-wu.ac.jp] |
| 22 | Completion Date | Last participant |
| 23 | Summary Results | N/A  The results of this trial will be disseminated through publications in relevant peer-reviewed journals |
| 24 | IPD sharing statement | Plan to share IPD: No |
