# Supplementary material for: A parallel-group randomized controlled trial of a culturally adapted, rumination-focused cognitive-behavioral therapy (RFCBT) guided self-help targeting repetitive negative thoughts in Japanese female university students – study protocol for the RESUME-CBT trial
Source: BMC Psychol. 2026 Feb 16;14:275. doi: 10.1186/s40359-026-04182-5 (PMC12947469; doi:10.1186/s40359-026-04182-5)
Supplement: Supplementary file 3 — Supplementary Material 3. Appendix 3. Description of the intervention. [file 40359_2026_4182_MOESM3_ESM.doc]

*Appendix 3. Description of the intervention in TIDieR format (Hoffman et al., 2014)*

| Item No. | Item | Description |
| --- | --- | --- |
| Brief Name | | |
| 1 | Provide the name or a phrase that describes the intervention | A therapist-guided, rumination-focused cognitive-behavioral therapy (RFCBT) self-help intervention designed for individuals with a high tendency toward rumination and worry. |
| Why | | |
| 2 | Describe any rationale, theory, or goal of the elements essential to the intervention | Young adult females in Japan are particularly vulnerable to depression and anxiety, with rumination recognized as a significant risk factor contributing to both the onset and maintenance of these conditions. In Western populations, RFCBT has demonstrated effectiveness in treating and preventing depression and anxiety by reducing the frequency of rumination. However, cultural adaptation has been crucial in optimizing its effectiveness. This study aims to address rumination among high-risk female Japanese university students through a culturally adapted, therapist-guided RFCBT self-help intervention. |
| What | | |
| 3 | Materials: Describe any physical or informational materials used in the intervention, including those provided to participants or used in intervention delivery or in training of intervention providers. Provide information on where the materials can be  accessed (such as online appendix, URL) | The self-help intervention consists of four structured modules, each presented as a separate workbook (16-28 pages).  *Module 1: “What is rumination?”*  This module explores repetitive negative thoughts (RNT) as a maladaptive coping strategy, introducing self-monitoring techniques to identify patterns and functions of RNT. To manage RNT effectively, it emphasizes learning from experience and implementing contingency plans (“If-Then” plans).  *Module 2: “Experimenting with ‘concrete thinking’”*  This module differentiates between concrete, specific, process-focused thinking and abstract, overgeneralized thinking styles. It incorporates experiential exercises, including the “Why-How experiment”, to facilitate this distinction. Additionally, it introduces contingency “If-Then” plans designed to promote a more concrete and adaptive thinking style.  *Module 3: “Fostering self-compassion”*  This module contrasts self-compassionate and self-critical self-talk, integrating experiential exercises to highlight their effects. Participants engage in activities that foster self-compassionate behaviors, and apply contingency “If-Then” plans to enhance compassionate self-talk and actions.  *Module 4: “Exploring absorbing memories”*  This module focuses on the re-experiencing of absorbing memories and the use of immersive activities as adaptive coping strategies. It includes contingency “If-Then” plans to facilitate absorption and encourages the incorporation of positive, engaging, absorbing activities into daily life. |
| 4 | Procedures: Describe each of the procedures, activities, and/or processes used in the intervention, including any enabling  or support activities | During the intervention, each participant will receive the workbooks sequentially, one at a time. Upon completing each workbook, they will engage in an individual face-to-face session with the trial therapist (approximately 15-40 minutes). These sessions will focus on consolidating acquired techniques and knowledge, reflecting on helpful techniques and their applications, and refining concrete plans for sustained use of effective strategies (including “If-Then” plans). Additionally, the sessions will emphasize the importance of repeated practice, addressing challenges and obstacles, and providing tailored support to facilitate ongoing progress. For each completed workbook, participants will be awarded a book voucher valued at 1,000 yen as compensation. |
| Who provided | | |
| 5 | For each category of intervention provider (such as psychologist, nursing assistant), describe their expertise, background,  and any specific training given | The therapist is a registered Certified Public Psychologist (CPP)/Clinical Psychologist (CP) in Japan, who has extensive experience in RFCBT (having completed 1- and 2-day workshops, and received individual and group supervision for face-to-face individual cases and self-help guides). |
| How | | |
| 6 | Describe the modes of delivery (such as face to face or by some other mechanism, such as internet or telephone) of the intervention and whether it was provided individually or in a group | A structured guided self-help format, supplemented by therapist guides delivered through individual face-to-face sessions. |
| Where | | |
| 7 | Describe the type(s) of location(s) where the intervention occurred, including any necessary infrastructure or relevant features | Nara Women’s University, Japan |
| When and How Much | | |
| 8 | Describe the number of times the intervention was delivered and over what period of time including the number of sessions,  their schedule, and their duration, intensity, or dose | The self-help intervention consists of four modules, with participants encouraged to complete each module within 1-2 weeks. |
| Tailoring | | |
| 9 | If the intervention was planned to be personalised, titrated or adapted, then describe what, why, when, and how | The self-help intervention follows a structured, module-based approach presented in a workbook format. However, during face-to-face sessions with the therapist, participants are encouraged to reflect on and reinforce the strategies they found most beneficial, ensuring their continued application. |
| Modifications | | |
| 10 | If the intervention was modified during the course of the study, describe the changes (what, why, when, and how) | N/A |
| How well | | |
| 11 | Planned: If intervention adherence or fidelity was assessed, describe how and by whom, and if any strategies were used to  maintain or improve fidelity, describe them | To ensure fidelity to the original RFCBT principles, the self-help intervention was translated and systematically adapted from the original RFCBT program, “MindReSolve” (Cook et al., 2019). |
| 12 | Actual: If intervention adherence or fidelity was assessed, describe the extent to which the intervention was delivered as planned | N/A |
