# Supplementary material for: A parallel-group randomized controlled trial of a culturally adapted, rumination-focused cognitive-behavioral therapy (RFCBT) guided self-help targeting repetitive negative thoughts in Japanese female university students – study protocol for the RESUME-CBT trial
Source: BMC Psychol. 2026 Feb 16;14:275. doi: 10.1186/s40359-026-04182-5 (PMC12947469; doi:10.1186/s40359-026-04182-5)
Supplement: Supplementary file 4 — Supplementary Material 4. Appendix 4. Documentation given to participants and consent form. [file 40359_2026_4182_MOESM4_ESM.doc]

**Instructions for Participants in the Study**

(*For Participation in “Promoting Resilience through Rumination-focused Cognitive Behavioral Therapy Self-Help Program for Female University Students - A Randomized Controlled Trial”*)

The following provides an overview of the research purpose and the specific methods of implementation for the study to which you are invited to participate.

Participation in this study is entirely voluntary. Your autonomy will be respected throughout the process. Should you choose not to participate, no adverse consequences will arise. Even after providing consent, you retain the right to refuse or withdraw your participation at any time without any negative implications.

This study has been reviewed and approved by the Nara Women’s University Ethics Review Committee for Research Involving Human Subjects (http://www.nara-wu.ac.jp/nwu/research/irb/index.html) and has received approval from the President of Nara Women's University (approval numbers 23-32, 23-59, 23-63, 24-55, 24-81).

Title of the study: “*Promoting Resilience through Rumination-focused Cognitive Behavioral Therapy Self-Help Program for Female University Students - A Randomized Controlled Trial*”

1. **Purpose and Significance of the Study**

The aim of this study is to investigate whether a self-help psychological program, presented in a workbook format, targeting “worry and ruminative thoughts,” can effectively promote problem-solving, reduce stress, and enhance psychological resilience.

Resilience, in psychological terms, refers to mental fortitude—the ability to adapt flexibly and recover from adversity and stressful situations encountered in daily life. It is not merely resistance to external stressors but the capacity to bounce back and regain composure, even when experiencing emotional challenges such as depression (much like a soft ball regaining its shape after being squeezed). Through this program, participants will learn various stress management techniques to apply in their daily lives, aimed at preventing negative thought patterns and reducing feelings of depression and anxiety.

The program is grounded in a psychological approach known as “Rumination-focused cognitive behavioral therapy,” developed in the United Kingdom. Research has demonstrated the effectiveness of this approach in alleviating depression and anxiety in young individuals. This study seeks to determine whether this approach is similarly effective for young people in Japan and whether it can be self-administered through a workbook format.

The program focuses on addressing worry and ruminative thinking, and we are seeking participants who regularly experience such thoughts but are not currently experiencing significant depression or anxiety, to take part in the program.

1. **Eligibility for Participation in this Study**

**(1) Who Can Participate**

Undergraduate and graduate students (ages 18-30) at Nara Women's University. Participants must score above a certain threshold on the scales of rumination and worrying tendency, which will be administered prior to participation in the study, and must demonstrate a tendency for ruminative thinking.

**(2) Who Cannot Participate**

This study is not designed to address mental health diagnosis. Therefore, individuals who score above a certain threshold on the depression scale, which will be administered prior to participation, or those who experience a strong ideation of death at the time of participation, and for whom professional care is deemed necessary, will not be included. In such cases, participants will be recommended to visit treatment and/or student support services. Additionally, individuals currently receiving psychiatric treatment, counseling, or psychological consultation are excluded from participation in this study.

1. **Methods of Conducting the Study**

**(1) Research Methods**

Participants who agree to join the study will first complete a screening questionnaire. Those who meet the specified criteria will be invited to participate. Participants will be randomly assigned to one of two groups:

1. Those who begin the program immediately.
2. Those who begin the program after an 8-week waiting period.

(a) Participants who participate in the program immediately

Participants will be provided with one workbook at a time as part of the program (four workbooks in total). For each of the four modules, you will complete the associated workbook within one week and apply the methods outlined in the workbook to your daily life.

The research administrator will send a questionnaire via email 4 weeks and 8 weeks after you begin the program. You are asked to respond truthfully regarding your condition during these periods.

After completing each workbook, please email the research therapist (narajo2024rfcbttherapist@gmail.com) and bring your completed workbook to your next appointment. During the appointment, an individual interview (30-60 minutes) will be conducted to discuss your impressions of the workbook and the lessons you have learned. Upon submitting your completed questionnaire, you will receive an acknowledgment and the next workbook.

(b) Participants who participate in the program after an 8-week waiting period

You will initially be placed on a waiting list. During the waiting period, no specific tasks are required, but you will need to complete questionnaires during and after the waiting period. After the 8 weeks, you will begin the program.

**(2) Location and Duration of the Study**

After completing each workbook, you will submit the completed questionnaire to the research therapist at a designated location on campus (Room D406 in Building D or Room E161 in Building E) and receive the next workbook. During this submission, you will be interviewed (30-60 minutes) to share your feedback on the workbook.

Each workbook takes approximately one hour to complete, but since the workbooks include plans and alternative coping strategies designed for real-life application, you will be expected to work on them over the course of one week.

The entire program, which includes completing all four workbooks, will take approximately 4 weeks. For those who begin after the 8-week waiting period, the program will take roughly 12 weeks to complete, including the waiting period.

**4. Persons Conducting the Research**

Principal investigator: Yusuke Umegaki (Associate Professor of Clinical Psychology, Graduate School of Life and Environmental Sciences)

Trial managers: Yui Kuroiwa (Master’s Course in Clinical Psychology, Graduate School of Humanities and Sciences)

Rina Honda (Master’s Course in Clinical Psychology, Graduate School of Humanities and Sciences)

**5. Expected Benefits and Burdens (Risks) of Participation in the Study**

The psychological program provided in this study will teach techniques to break free from negative ruminative thinking and improve coping strategies for stressful situations. Therefore, participation may offer the benefit of enhancing your ability to manage stress effectively and help you overcome negative thought patterns.

However, the program will also address challenging situations and stressors, and participants will be required to complete questionnaires assessing their levels of depression and anxiety at the time of participation. If you find that this is becoming overwhelming, please contact the principal investigator or the trial manager immediately.

**6. Handling of Personal Information**

Data and personal information collected will be used solely for the purpose of conducting and verifying this research. Data will be stored either online or on an external storage medium (external hard drive) using a computer with sufficient safeguards to prevent information leakage, and will be kept in a locked laboratory to prevent loss or theft. Identifiable information will always be stored separately, made linkable, and anonymized.

Personal information will be handled with the utmost care and rigorously managed to prevent unauthorized access. Data and personal information will be stored for five years (10 years for paper records) after the completion of the study, after which it will be disposed of securely to prevent any potential leakage of personal information. The consent form submitted to us will be retained by the principal investigator (Yusuke Umegaki) and will be securely disposed of after the research period.

**7. Disclosure of Research Materials and Information**

Upon request, we will disclose information regarding the research plan and methods of this study, to the extent that it does not compromise the protection of participants’ personal information or undermine the originality of the research. If you have any questions about the study, please feel free to contact the principal investigator or the trial manager at any time.

**8. Publication of Research Results**

We plan to publish the results of this research in the form of the theses by the trial managers, as well as through conference presentations and academic papers at relevant conferences. When publishing the results, we will carefully protect the privacy of participants. No personally identifiable information will be included in any publications.

**9. Free Will Consent and Freedom to Withdraw Consent**

Participation in this research is entirely voluntary. You will not face any disadvantages if you choose not to participate. Even after providing consent, you may withdraw your participation at any time without facing any negative consequences. If you wish to withdraw, please complete the “Consent Withdrawal Form” attached to the last page of this instruction manual and hand it to the principal investigator or trial manager. In this case, any data you have provided will be discarded and will not be used for further research. However, in cases where research results have already been published in an article or other publication, or when data and other information have been fully anonymized and cannot be identified, the data may not be discarded.

**10. Sources of Fundings for this Research**

The funding for this research will be provided by a Grant-in-Aid for Scientific Research from the Japan Society for the Promotion of Science (JSPS), as well as research funds from Nara Women's University.

**11. Conflicts of Interest*** **in Relation to the Research**

There are no conflicts of interest related to this research, such as involvement with companies or any financial interests that may influence the results of the research or the protection of participants.

*Definition of “Conflict of Interest”:

A conflict of interest occurs when a third party may raise concerns that a financial or other interest relationship with a company or entity could impair the fair and proper judgment required to conduct the research.

**12. Compensation (Rewards) for Research Participants**

A library card worth 1,000 yen will be provided to each participant for every completed workbook.

**13. Ownership of Intellectual Property Rights**

Intellectual property rights may arise from the results of this research; however, these rights will belong to Nara Women's University, the institution overseeing this research, and not to the participants.

**14. Supplementary**

This study will utilize the same program that was conducted at Nara Women's University during the academic years 2017-2018 and 2021-2022.

For inquiries regarding the research and the content of the research plan, please contact:

Principal investigator: Yusuke Umegaki (Associate Professor of Clinical Psychology, Graduate School of Life and Environmental Sciences, Nara Women's University)

Trial manager: Yui Kuroiwa / Rina Honda

E-mail: narajo2024rfcbttherapist@gmail.com

If you agree to participate in this research after carefully reading and understanding the above information, please sign and date the attached “Consent for Research Participation Form” and hand it to the person in charge.

**Consent for Research Participation Form**

Dear Yusuke Umegaki, Principal Investigator,

I have received an explanation of the following items regarding the research project entitled “*Promoting Resilience through Rumination-focused Cognitive Behavioral Therapy Self-Help Program for Female University Students - A Randomized Controlled Trial*”. I have indicated my understanding of the items by placing a ✓ in □ by myself.

□ 1. Purpose and Significance of the Study

□ 2. Eligibility for Participation in this Study

□ 3. Methods of Conducting the Study

□ 4. Persons Conducting the Research

□ 5. Expected Benefits and Burdens (Risks) of Participation in the Study

□ 6. Handling of Personal Information

□ 7. Disclosure of Research Materials and Information

□ 8. Publication of Research Results

□ 9. Free Will Consent and Freedom to Withdraw Consent

□ 10. Sources of Fundings for this Research

□ 11. Conflicts of Interest in Relation to the Research

□ 12. Compensation (Rewards) for Research Participants

□ 13. Ownership of Intellectual Property Rights

I have indicated my consent to the release of my voice recordings for this research by marking a ✓ in the appropriate box below.

□ I do not consent to the release.

□ I consent to the release solely for academic purposes by the researcher.

□ I consent to the disclosure under the following conditions:

□ I agree to the inclusion of personally identifiable information.

□ I consent only if individuals cannot be identified.

□ Other (Please specify any special requests below):

After confirming these items, I agree to participate in this research.

　　　　　　　　Year: Month: Day:

　　Participant’s Signature:

**Consent Withdrawal Form**

Principal Investigator: Dr. Yusuke Umegaki,

I hereby withdraw my consent to participate in the study “*Promoting Resilience through Rumination-focused Cognitive Behavioral Therapy Self-Help Program for Female University Students - A Randomized Controlled Trial*”, for which I previously signed the consent form.

　　　Year: Month: Day:

Participant’s signature:

(For Principal Investigator use)

I acknowledge receipt of the Consent Withdrawal Form for this study.

　　　　Name (self-signed):

　　　　Affiliation:

　　　　Position Title:
